# Bacteriophage Protects Against *Aerococcus viridans* Infection in a Murine Mastitis Model

**DOI:** 10.3389/fvets.2020.00588

**Published:** 2020-08-28

**Authors:** Hengyu Xi, Dali He, Dong Li, Shan-shan Liu, Gang Wang, Yalu Ji, Xinwu Wang, Zijing Wang, Lanting Bi, Rihong Zhao, Hao Zhang, Li Yang, Zhimin Guo, Wenyu Han, Jingmin Gu

**Affiliations:** ^1^Key Laboratory of Zoonosis Research, Ministry of Education, College of Veterinary Medicine, Jilin University, Changchun, China; ^2^Department of Immunology, College of Basic Medical Sciences, Jilin University, Changchun, China; ^3^Department of Chinese Journal of Veterinary Science, Jilin University, Changchun, China; ^4^Department of Clinical Laboratory, The First Hospital of Jilin University, Changchun, China; ^5^Jiangsu Co-Innovation Center for the Prevention and Control of Important Animal Infectious Disease and Zoonose, Yangzhou University, Yangzhou, China

**Keywords:** bacteriophage, *Aerococcus viridans*, bovine mastitis, murine model, phage therapy

## Abstract

Bovine mastitis, an inflammatory disease that occurs frequently in early lactation or the dry period, is primarily caused by bacterial infections. There is growing evidence that *Aerococcus viridans* (*A. viridans*) is becoming an important cause of bovine mastitis. The treatment of bovine mastitis is primarily based on antibiotics, which not only leads to a large economic burden but also the development of antibiotic resistance. On the other hand, bacteriophages present a promising alternative treatment strategy. The object of this study was to evaluate the potential of a previously isolated *A. viridans* phage vB_AviM_AVP (AVP) as an anti-mastitis agent in an experimental *A. viridans*-induced murine mastitis model. *A. viridans* N14 was isolated from the milk of clinical bovine mastitis and used to establish a mastitis model in mice. We demonstrated that administration of phage AVP significantly reduced colony formation by *A. viridans* and alleviated damage to breast tissue. In addition, reduced inflammation was indicated by decreased levels of inflammatory cytokines (TNF-α, IL-1β, and IL-6) and myeloperoxidase (MPO) activity in the phage-treated group compared to those in the phosphate buffered saline (PBS)-treated group. To the best of our knowledge, this report is the first to show the potential use of phages as a treatment for *A. viridans*-induced mastitis.

## Introduction

Bovine mastitis is an inflammatory response primarily caused by infection of the udder tissue, which has a serious impact on the health of dairy cows and the quality of their milk (1). Bovine mastitis results in prevalent and large-scale financial losses in the global dairy industry (2). With advances in the modern dairy industry, farmers have been constantly seeking low-cost and effective ways to minimize the damage caused by mastitis. Although optimizing the milking process, antibiotic use and disposal of infected animals decreased this damage, these methods led to an increase in the cost of milk production. Even more worrying is the emergence of drug resistance and the contamination of residues in milk due to the overuse of antibiotics (2, 3). As a result, restrictions on antibiotics and new methods for combating resistant bacteria are urgently needed globally (3–5), making the development of new drugs and the discovery of new treatments even more important.

Mastitis is considered to be primarily caused by major pathogens, such as *Streptococcus agalactiae, Streptococcus uberis* and *Staphylococcus aureus*, while infections by these pathogens have decreased in recent decades due to the implementation of specific control strategies (6, 7). The incidence of mastitis caused by minor pathogens, including *Corynebacterium* spp. and coagulase-negative staphylococci, is increasing (8, 9). In this case, *A. viridans* bacteria are increasingly becoming a common pathogen causing mastitis (10, 11). *A. viridans* is a Gram-positive opportunistic pathogen that belongs to the family *Aerococcaceae* and is widely distributed in the environment, that is, air, water, and soil (12). In 2004, Zadoks et al. detected *A. viridans* in 50% of bulk milk samples from 48 dairy farms in the USA (13). The isolation rates of *A. viridans* in subclinical mastitis in China reported by Liu et al. (14) and Sun et al. (10) were 6.1 and 16.67%, respectively. In addition, in a 2015 report in Japan, the incidence of *A. viridans* was 8% in 478 cases of clinical mastitis, while the incidences of *Streptococcus* spp. and coagulase-negative *Staphylococcus* were 9.2 and 3.1%, respectively (15). In recent studies, Liu et al. confirmed that virulent *A. viridans* had the ability to adhere and invade bMECs and possessed strong cytotoxicity (16). However, the mechanism by which *A. viridans* causes mastitis has not been elucidated. More disturbingly, antibiotic-resistant strains of *A. viridans* are also increasing (14, 17).

Bacteriophage therapy may represent a promising alternative to antibiotics due to its ability to specifically infect and kill bacteria. The treatment of humans by bacteriophages has been achieved in Poland, Georgia and Russia and has a long history (18). According to the data published by the Phage Therapy Center of the Hirszfeld Institute of Immunology and Experimental Therapy in Wroclaw, 35–50% of patients received positive feedback after phage treatment (19). In addition, bacteriophages also have useful application in veterinary clinics (20). Researchers have reported that phages can be used for the treatment of infections caused by multiple drug-resistant bacteria, such as *Enterococcus faecium* (21), *Escherichia coli* (22), and *Pseudomonas aeruginosa* (23). In previous studies, phages were effective in treating *S. aureus* and *E. coli*-induced mastitis models in mice (24, 25). These results indicate that phage therapy is feasible, but there is no report describing the use of phage therapy in the treatment of mastitis caused by *A. viridans* to date.

In our previous study, the first phage of *A. viridans* was isolated, and its general biological characteristics and genomic characteristics were analyzed (26), indicating that it is safe at the genome level and has potential for application. In this study, the murine mastitis model was established by *A. viridans*, and the therapeutic effect of phage vB_AviM_AVP (AVP) on the murine mastitis model was evaluated.

## Materials and Methods

### Ethics Statement

All animal studies were conducted according to the National Guidelines for Experimental Animal Welfare (Ministry of Science and Technology of China, 2006) and were approved by the Animal Welfare and Research Ethics Committee at Jilin University (Permit Number: pzpx20181227051). In the experiment, the animals were treated humanely, and every effort was made to reduce the suffering of the animals.

### Bacterial Strains and Culture Conditions

Bacterial strains M13-1, N14, N15, M6-1, M19-1, and P_1−1_F_2_ were all isolated from milk samples of dairy cows with clinical mastitis. Milk samples were cultured overnight on blood agar plates, and circular colonies with off-white color that could form α-hemolysis were selected from the mixture containing various bacteria. Next, the suspected colonies were subjected to pure culture and a series of identifications to confirm whether they were *A. viridans*. The initial identification was based on the colony morphology, hemolytic reaction, and microscopic morphology followed by PCR with a pair of specific primers F (5′-GTGCTTGCACTTCTGACGTTAGC-3′) and R (5′-TGAGCCGTGGGCTTTCACAT-3′) (27). The PCR amplification products were sequenced and aligned using Nucleotide-Blast in the NCBI database. CZ4b-3 and Aer-1 ~ Aer-6 were isolated from pigs with clinical diseases and were generously donated by Nanjing Agricultural University and Jilin Agricultural University (28), respectively. *Aerococcus urinae* (*A. urinae*) ATCC 51268 was obtained from the American Type Culture Collection (ATCC). All the strains were cultured at 37°C on TSA (trypticase soy agar, Sigma, Shanghai, China) containing 5% defibrinated sheep blood. Subsequently, the bacteria were cultured routinely at 37°C in brain-heart infusion (BHI, Becton-Dickinson, Franklin Lakes, NJ, USA) broth and stored in 30% glycerol at 80°C.

### Stability of Phage vB_AviM_AVP at Different pH Values and Temperatures

The bacteriophage AVP was isolated by co-cultivation of host bacteria and sewage samples in BHI medium as reported previously (26), previous studies have shown that AVP has a latent phase of 15 min and a burst size of 139 PFU/cell, and its optimal multiplicity of infection is 0.001 (Figure S2). To determine whether AVP has potential as a therapeutic phage, further stability determination is necessary.

To determine the pH stability of the phage, the titres were determined by the double-layer agar method after incubating the same concentration of phage suspensions in SM buffer (pH: 1-12) at 37°C for 2 h. For the detection of bacteriophage temperature stability, 10^8^ PFU (plaque forming unit) of bacteriophage suspensions were incubated at 25°C, 40°C, 50°C, 60°C, and 70°C for 100 min, and samples were taken at 20-min intervals for titer determination.

### Determination of Host Range of Phage AVP

In this experiment, a total of 14 strains of *A. viridans* and 1 strain of *A. urinae* were used to detect the host range of phage AVP. CsCl density gradient centrifugation was used for phage purification, as previously described (26). Ten microlitres of purified phage AVP was dropped onto plates that were covered with bacterial lawns of different *Aerococci* strains. The host range was characterized by spot test and assayed at 37°C for 18–24 h.

### *A. viridans*-Induced Murine Mastitis Model

Specific-pathogen-free 6–8-week-old female BALB/c pregnant mice (18–20 d old) were purchased from the Experimental Animal Center of Jilin University. During the experiment, the mice were kept in the animal room with a temperature-controlled and light-dark (light: 8:00–20:00) cycle. Feed and fresh water were available *ad libitum*.

The murine mastitis model was established according to a previous description with minor modifications (29). Briefly, female mice at 7–10 days of lactation were used for mammary gland inoculation, and the pups were separated from lactating mice 1–2 h before the experiment. A mixture of ketamine (87 mg/kg) and xylazine (13 mg/kg) was used for intraperitoneal anesthesia in mice before challenge (29).

### Bacteriophage Therapy in a Murine Mastitis Model

In this study, a total of 36 lactating BALB/c mice were randomized into 6 groups: (1) Bacteria-PBS group; (2) Bacteria-AVP-L group; (3) Bacteria-AVP-M group; (4) Bacteria-AVP-H group; (5) Control group; and (6) Safety-Control group. The teat tips of the fourth pair of mammary glands were cut off, and 50 μL of *A. viridans* N14 suspension (5 × 10^6^ CFU/gland) was delivered into both the left and right mammary glands of 4 groups of mice through the exposed teat canal with a 32-gauge blunted needle. The other 2 groups were the Control group (50 μL sterile PBS/gland) and the Safety-Control group only with 50 μL phage AVP (5 × 10^7^ PFU/gland) intramammary injection.

To determine the effect of phage AVP against murine mastitis, 4 h after *A. viridans* challenge, the above 4 groups of infected mice were intramammarily inoculated with 50 μL of sterile PBS (Bacteria-PBS group), low dose (5 × 10^5^ PFU/gland, Bacteria-AVP-L group), medium dose (5 × 10^6^ PFU/gland, Bacteria-AVP-M group), and high dose (5 × 10^7^ PFU/gland, Bacteria-AVP-H group) of purified phage AVP. The other two groups of mice were not treated. All mice were euthanized by cervical dislocation 24 h after challenge, and the mammary glands were aseptically removed. One portion of weighed mammary glands was resuspended in sterile PBS, and sterile mortars and motor-driven Teflon pestles (JinTai, Changchun, China) were further used for homogenization. To determine the bacterial load in the mammary glands, 100 μL of serially diluted homogenates were counted on BHI agar plates. The PFU of all phage-treated groups was obtained by centrifuging the mammary gland homogenate at 4°C for 5 min (12,000 × g) and filtering and counting with 100 μL of serially diluted filtrate on double-layer agar plates. The limits of detection (LOD) were CFU or PFU that could not be detected by directly counting the original homogenate. The other portion of the mammary glands was used for histopathological analysis, as well as the detection of MPO and inflammatory cytokines.

### Histological Evaluation of Breast Tissues

Histopathological analysis of mammary gland tissues of different treatment groups was performed. Briefly, all mice were euthanized at 24 h post-inoculation, and mammary glands were removed. Tissue samples from mammary glands were immediately fixed in 4% paraformaldehyde and further embedded in paraffin and sectioned. In addition, pathological damage to the breast tissue was evaluated by haematoxylin and eosin (H&E) staining with optical microscopy.

### MPO (Myeloperoxidase) Activity Detection and Inflammatory Cytokine Assay

MPO is a functional and activation marker of neutrophils (30), and its activity was detected as follows. Briefly, aseptically collected mammary tissues were weighed and added to HEPES (containing 0.1 mg/mL of STI) buffer solution at 0.2 g/mL. After grinding (50 Hz, 10 s interval) for 20 min, the homogenate was centrifuged at 4°C for 30 min at 12,000 × g. Next, an equal volume of 0.5% CTAC was added to the precipitate, which was ground and centrifuged again. Finally, the collected 5-fold diluted supernatants were measured for changes in absorbance at 450 nm using a 96-well plate reader to evaluate activity.

Enzyme-linked immunosorbent assay (ELISA) kits for detecting TNF-α, IL-1β, and IL-6 in the mammary glands were obtained from Biolegend (San Diego, CA, USA), and the operation was performed according to the manufacturer's instructions. In brief, breast tissues were ground (50 Hz, 10 s interval) for 20 min after adding sterile PBS buffer at 0.2 g/mL. After centrifugation at 4°C for 20 min at 12,000 × g, the supernatant was used for the detection of inflammatory cytokines.

### Statistical Analysis

All statistical analyses were performed using SPSS version 13.0 software (SPSS, Inc., Chicago, IL, USA). The significance of experimental data *in vivo* was determined with a nonparametric Kruskal–Wallis test followed by pairwise comparisons. ^*^P < 0.05, ^**^P < 0.01, and ^***^P < 0.001 were considered to indicate significance. The error bars represent the standard deviation (SD) of the mean.

## Results

### Isolation and Identification of *A. viridans*

The strains isolated from clinical mastitis samples can form typical alpha haemolysis after culture on blood agar plates, and the paired or clustered Gram-positive cocci were observed under the microscope. The 16S rRNA gene fragments of *A. viridans* strains were amplified using PCR, and the fragments measured ~540 bp according to gel electrophoresis (Figure S1) and sequencing. Nucleotide-Blast analysis of sequencing results showed that they have 97–100% identity with *A. viridans* in the GenBank database.

### pH and Thermal Stability

**Figure 1:**
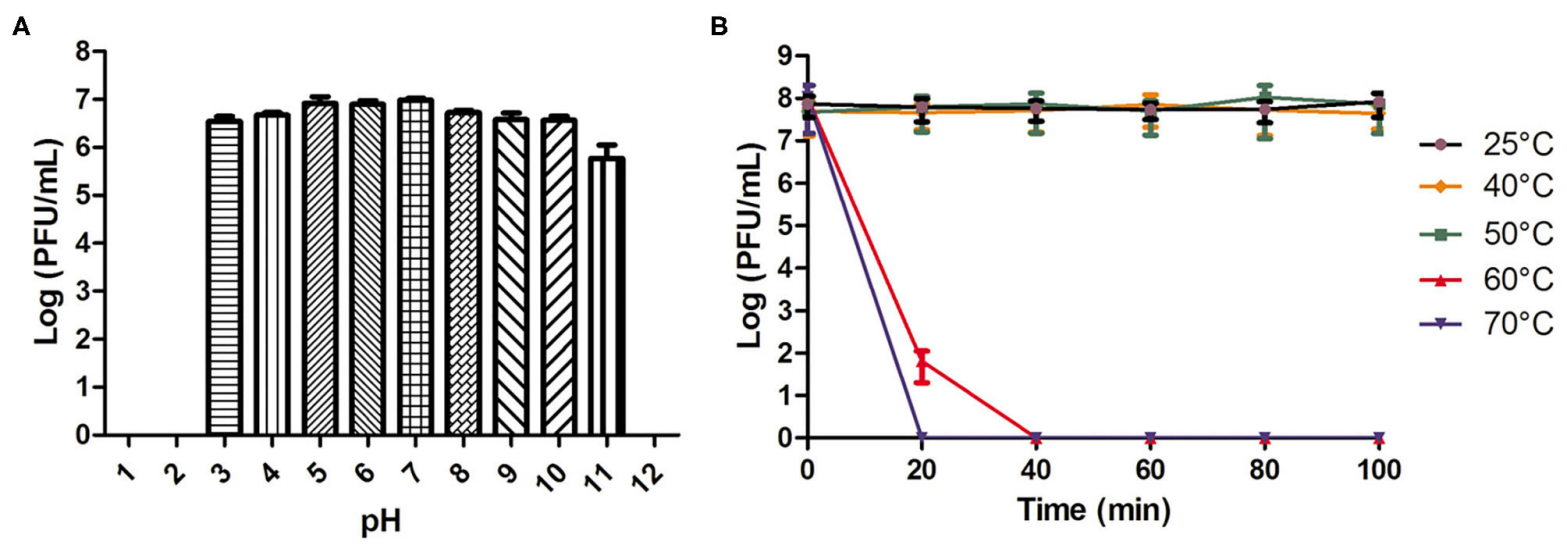
pH and temperature stability of phage AVP. **(A)** The stability of phage AVP in SM buffer at different pH values for 2 h. **(B)** Stability of phage AVP performed at 25°C, 40°C, 50°C, 60°C, and 70°C during 100 min. The Y-axis shows the log of plaque-forming units per milliliter (PFU/mL). Each assay was performed in triplicate, and the values represented are the means ± SD. N.D. not detected.

As shown in Figure 1A, phage AVP showed pH stability. There was no change in the phage titer after a 2-h incubation in SM buffer (pH 3-11). However, the phage activity was completely lost when the pH was lower than 3 or higher than 11. The phage activity at different temperatures is shown in Figure 1B. When the temperature was 25°C, 40°C, and 50°C, the phage titer showed no drop within 100 min. However, at 60°C and 70°C, the phage completely lost its activity at 40 and 20 min, respectively.

### Host Range of Phage AVP

As shown in Table S1, of all 15 strains tested, in addition to the host strain AV-X1 (isolated from pigs), AVP can also lyse 3 strains of *A. viridan* isolated from pigs and 4 strains of *A. viridan* isolated from bovines. However, AVP has no lytic activity against *A. urinae* ATCC 51268.

### Phage AVP Reduces Bacterial Load in Mammary Glands

**Figure 2:**
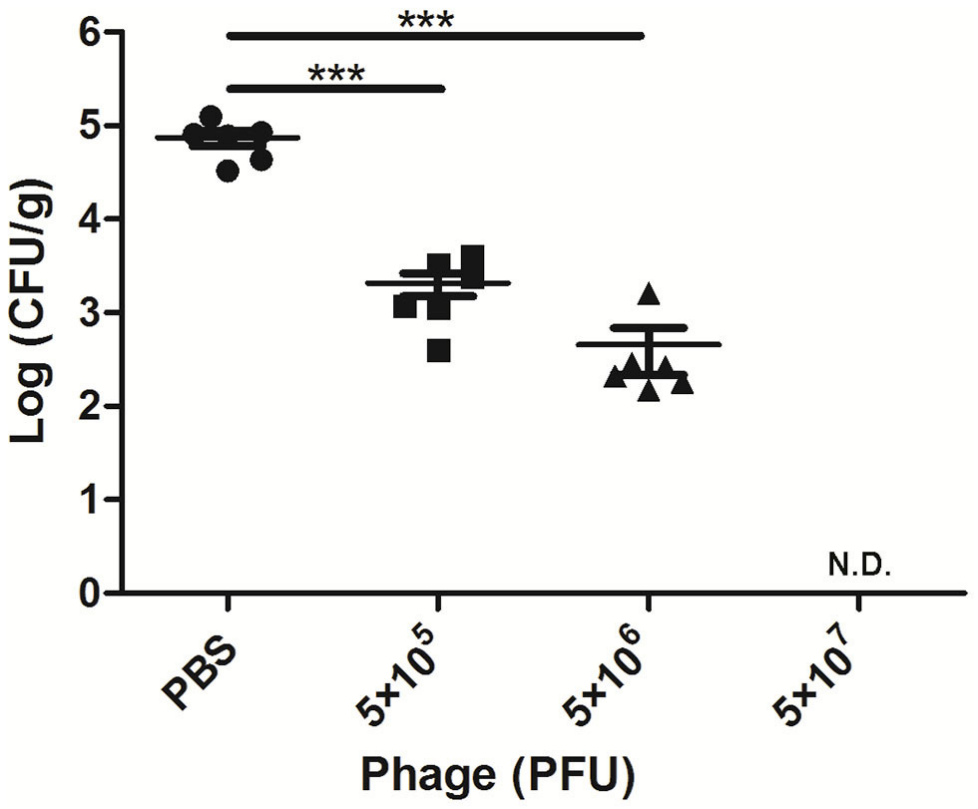
Bacterial load in the mammary glands of mice. The bacterial load in the mammary glands of mice in different treatment groups after 24 h of *A. viridans* challenge was detected. The mice infected with 5 × 10^6^ CFU of *A. viridans* N14 were treated with different doses of phage or PBS. At 24 h post-infection, ten-fold serially diluted mammary gland homogenates were plated to count bacterial CFU. Each icon on the figure corresponds to an individual gland. All data are shown as the mean ± SD. N.D. not detected. *P < 0.05, **P < 0.01, ***P < 0.001 compared to PBS treated group.

After 24 h of *A. viridans* challenge, a bacterial load of nearly 7.4 × 10^4^ CFU/g was detected in the mammary glands of the infected mice treated with PBS (Figure 2). When the mice were treated with AVP 4 h after challenge, the number of colonies in the mammary glands was significantly reduced (*P* < 0.001). The bacterial loads of the Bacteria-AVP-L group and Bacteria-AVP-M group were 2.1 × 10^3^ CFU/g and 4.5 × 10^2^ CFU/g, respectively. In the high-dose phage-treated mice, no colonies were detected in the mammary glands.

### Titer of Phage AVP in Mammary Glands After Therapy

**Figure 3:**
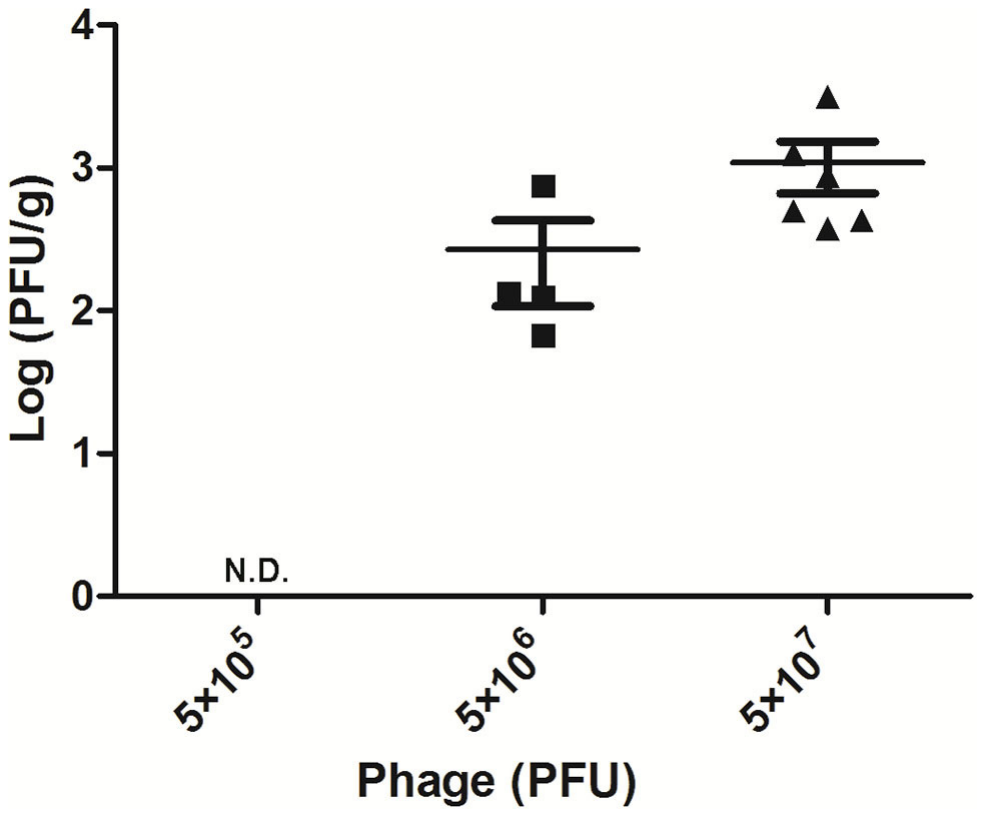
Mammary gland PFU post phage treatment. The phage counts of all the Bacteria-AVP groups were observed in mammary glands. The mammary glands of mice with different doses of phage treatment were homogenized and filtered at 24 h post-infection. The double-layer agar plate method was used to detect PFU in the mammary gland. Each icon on the figure corresponds to an individual gland. All data are shown as the mean ± SD.

To detect the phage titer in the mammary glands treated with different doses of AVP 24 h after challenge, the double-layer plate method was used to count phage in their tissue homogenates. No phage was detected in the Bacteria-AVP-L group. In contrast, phage remained in the mammary glands of the Bacteria-AVP-M group (1.8 × 10^2^ PFU/g) and the Bacteria-AVP-H group (8.7 × 10^2^ PFU/g) after 24 h (Figure 3).

### Effects of Phage AVP on *A. viridans*-Induced Mastitis Histopathological Changes

**Figure 4:**
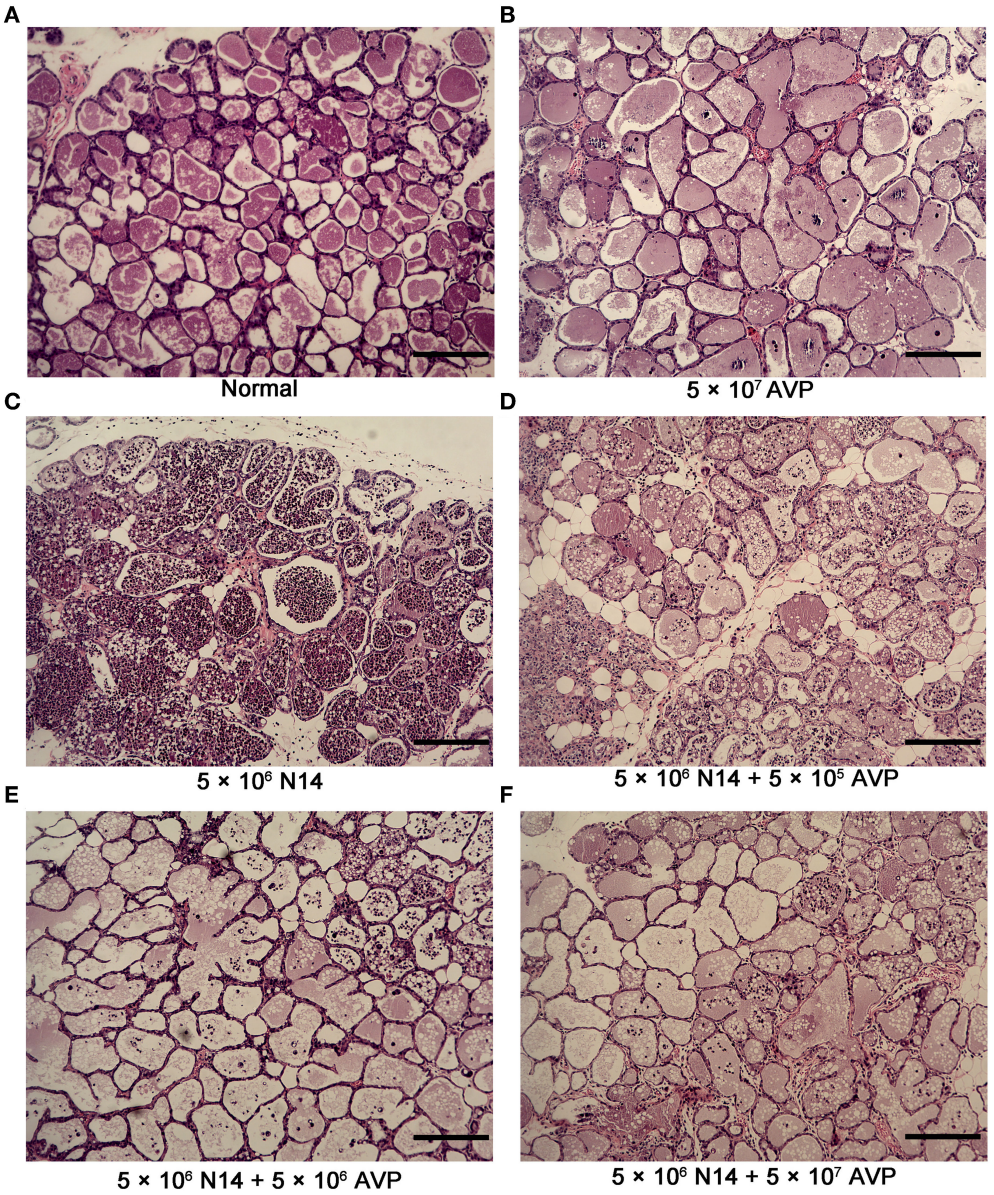
Histopathological examination of murine mammary glands. At 24 h post-inoculation of *A. viridans* N14 with or without AVP treatment, the mammary glands were removed for histopathological analysis. Representative H&E-stained mammary tissues from each group are shown (100×). **(A)** The Control group (Normal), **(B)** Safety-Control group (5 × 10^7^ AVP), **(C)** Bacteria-PBS group (5 × 10^6^ N14), **(D)** Bacteria-AVP-L group (5 × 10^6^ N14 + 5 × 10^5^ AVP), **(E)** Bacteria-AVP-M group (5 × 10^6^ N14 + 5 × 10^6^ AVP), **(F)** Bacteria-AVP-H group (5 × 10^6^ N14 + 5 × 10^7^ AVP). Scale bar = 200 μm.

To investigate the protective effect of phage AVP on *A. viridans*-induced mastitis in mice, H&E staining was used to analyse the pathological changes of the mammary glands (Figures 4A–F). Histopathological analysis showed that the control group had intact acinar walls and no inflammatory cell infiltration, showing the normal structure of breast cells (Figure 4A). In contrast, the acinar integrity of the Bacteria-PBS group was destroyed, and the acinar wall was thickened, which was accompanied by a large amount of inflammatory cell infiltration (Figure 4C). After phage therapy, the damage to the acinar structure caused by *A. viridans* was considerably weaker than that of the Bacteria-PBS group, and neutrophil infiltration was alleviated (Figures 4D–F). However, intact structure and no typical pathological damage were found in the Safety-Control group, indicating the safety of phage AVP application in this study.

### Effects of Phage AVP on MPO Activity and Inflammatory Cytokines in a Murine Mastitis Model

**Figure 5:**
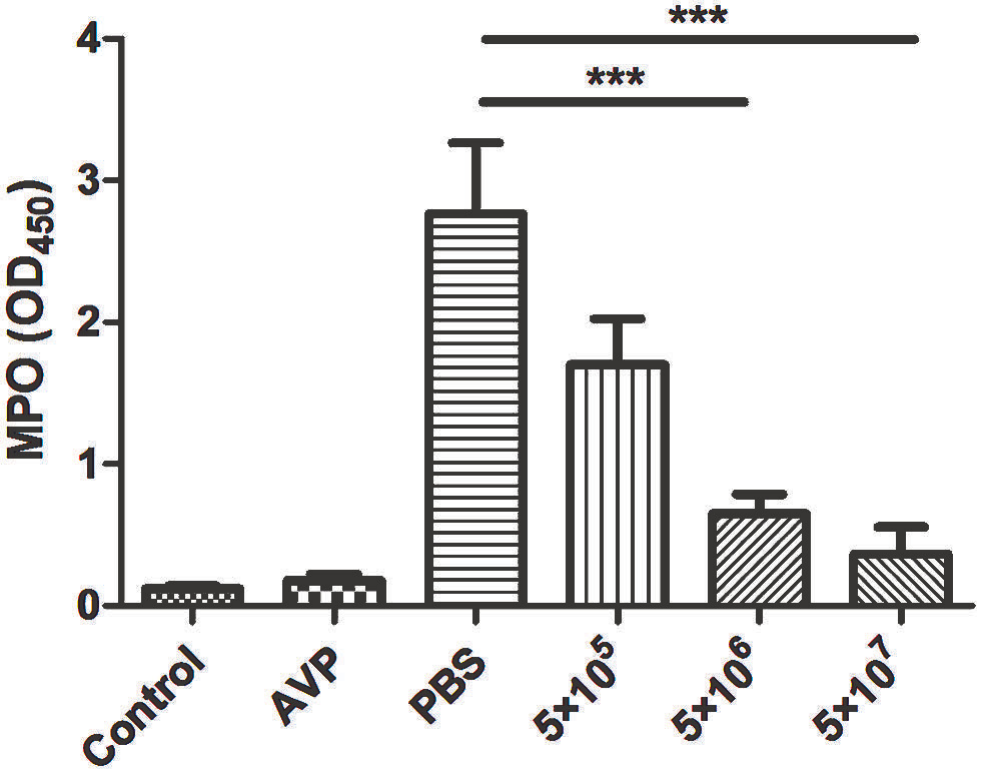
Effect of phage AVP on MPO activity in *Aerococcus viridans*-induced mastitis. MPO activity was measured in the mammary glands of different treatment groups at 24 post-inoculation. The mammary gland tissues of the healthy mice were used as controls. All data are shown as the mean ± SD (*n* = 6 each group). *P < 0.05, **P < 0.01, ***P < 0.001 compared to PBS treated group.

The inflammation level was assessed indirectly based on the level of the neutrophil marker MPO. After *A. viridans* infection, the accumulation of neutrophils in the mammary glands of the Bacteria-PBS group was significantly elevated (Figure 5). In contrast, MPO activity significantly decreased and approached the level of the control group after medium- and high-dose phage treatment (Figure 5, *P* < 0.001). Furthermore, there was no significant difference between the Control group and the Safety-Control group, which corresponded to the pathological changes.

**Figure 6:**
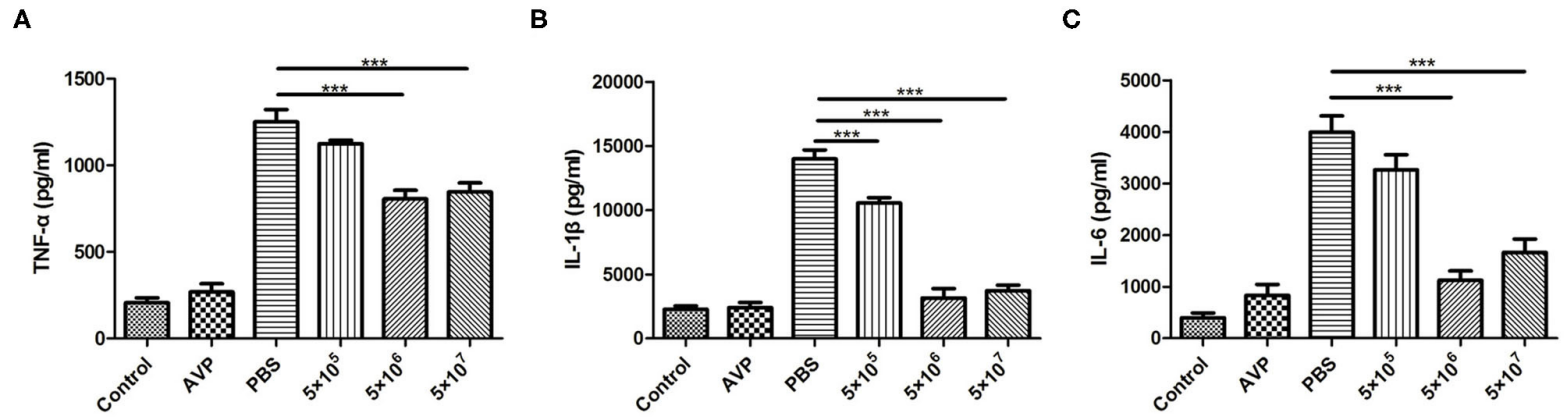
Cytokine levels in the mammary gland. At 24 h post-inoculation, the levels of pro-inflammatory cytokines in all mammary glands were measured with an enzyme-linked immunosorbent assay. The levels of TNF-α **(A)**, IL-1β **(B)**, and IL-6 **(C)** in murine mammary gland tissue of all treatment groups were determined. The tissues of the healthy mice served as controls. All data are shown as the mean ± SD (*n* = 6 each group). *P < 0.05, **P < 0.01, ***P < 0.001 compared to PBS treated group.

Inflammatory cytokines in the breast were also measured. As shown in Figure 6, the levels of TNF-α, IL-1β, and IL-6 after phage treatment were significantly lower than those of the Bacteria-PBS group (*P* < 0.001). In particular, the levels of IL-1β and IL-6 in the medium-dose and high-dose phage-treated groups were close to those in the control group. Similarly, the Safety-Control group did not show a significant increase in inflammatory cytokine levels compared to the Control group.

## Discussion

The main bacteria causing mastitis in dairy cows are *Streptococcus dysgalactiae, Streptococcus uberis, S. agalactiae, S. aureus*, and *E. coli* (31, 32); the latter three have been identified and studied by animal models (33–35). In particular, some studies have used phages to treat a mouse mastitis model caused by *S. aureus* and *E. coli*, which have shown therapeutic effects *in vivo* (25, 36, 37), indicating that the application of phages is safe and effective. Although the currently reported efficacy of bacteriophages on mastitis cows is absent or highly limited with a curation rate of only 16.7% (38, 39), which may indicate that more research is needed to investigate the complex interactions of phages and bacteria *in vivo*.

Bovine mastitis caused by *A. viridans* could not be overlooked. It is easy to mistake this bacterium for *Staphylococcus* or *Streptococcus* (40, 41), resulting in misjudgment and mistreatment. Misidentifying the bacteria will also have a strongly negative effect for phage therapy against *A. viridans*, considering phage species specificity. In fact, the mastitis caused by *A. viridans* is more serious than previously known. Additionally, with the long-term and large-scale use of antibiotics in the dairy industry, the problem of antibiotic resistance has become increasingly serious. The same problem is observed in the case of *A. viridans*, which has developed resistance to most of the commonly used antibiotics usually used for Gram-positive bacteria (14, 42, 43). There have been reports that *A. viridans* is even resistant to vancomycin and other back-up antibiotics (17, 44). Therefore, the use of antibiotics for the infection of potentially drug-resistant *A. viridans* will be limited. In addition, the application of antibiotics will cause the residue of antibiotics to exist in milk in a certain period of time, which will bring further losses to farmers and other unknown health risks to milk consumers. In contrast, phages are specific against certain strains of bacteria, and there are no reports about their harmful effects on both humans and animals (45).

In this work, we used a murine model of *A. viridans* induced mastitis, which showed typical symptoms of bovine mastitis as reported before (29). To date, AVP isolated in our earlier stage is the only reported bacteriophage that infects *A. viridans*. To verify the potential of its therapeutic value, we used AVP to treat the mastitis model. Our results showed that AVP can effectively remove *A. viridans* in the murine mastitis model, and the bactericidal effect of the phage is not affected by other factors *in vivo*. Comprehensive evaluation of pathological tissue sections, MPO activity, and inflammatory factor levels showed that phage treatment significantly improved the inflammation of breast tissue. These results are similar to the reported therapeutic effect of phage therapy on murine mastitis caused by other bacteria (25, 37), which further indicates the effectiveness of phage therapy in the treatment of mastitis.

This experiment is a therapeutic study of bacteriophages in mice infected with *A. viridans*. It is necessary to conduct research on the preventive effect of bacteriophages on mastitis. In future work, more strains, different times of phage administration and long-term follow-up will be involved to conduct in-depth research on the efficacy of phages; in particular, bona fide studies in dairy cattle are necessary. In addition, the residual *A. viridans* in faces, bedding materials and utensils are also responsible for bovine infection (15). It is conceivable that the bacteriophage AVP can be used as a spray to disinfect the farm environment and thus reduce infections by *A. viridans*, and AVP could play this effective preventive role based on its stability at different pHs and temperatures.

It must be acknowledged that phages are highly specific, which is an advantage and a limitation. Therefore, the selection of appropriate phages is the key factor for prophylactic or therapeutic applications. Because phage therapy belongs to precise and personalized therapy, it is necessary to specifically select appropriate phages for the prevention and treatment of a specific farm or patient. On the other hand, phages still exist in the mammary gland 24 h post-inoculation in this experiment, which is conducive to the further elimination of residual bacteria. Longer observation periods may enable further reduction of colonies and improvement of inflammation; nevertheless, the risk of bacterial resistance is also increased. However, both of the above disadvantages can be compensated for by phage cocktail therapy (46). In addition, mastitis in the clinic is likely to be caused by the coinfection of multiple bacteria; therefore, phages of various bacteria may need to be combined into a cocktail to achieve better prevention and treatment effects. Overall, phage use is indeed safe and promising, but there are a number of challenges that remain to be addressed.

## Conclusions

In this study, the murine mastitis model was established by using *A. viridans*. *A. viridans* infection caused significant pathological damage to the mammary gland tissue of mice. AVP can effectively eliminate the *A. viridans* that causes mastitis in mice. In addition, the application of AVP can effectively ameliorate the pathological damage to the mammary gland caused by *A. viridans* infection and the subsequent development of inflammation, as indicated by the histological analysis of mammary gland tissue and the measurement of MPO activities and pro-inflammatory cytokines. In summary, AVP has shown pharmaceutical potential in the treatment of mastitis caused by *A. viridans*. The results of this study indicate that bacteriophages are promising candidates for controlling bacterial infection.

## Data Availability Statement

All datasets presented in this study are included in the article/Supplementary Material.

## Ethics Statement

The animal study was reviewed and approved by Animal Welfare and Research Ethics Committee at Jilin University.

## Author Contributions

WH and JG contributed to conception and design of the study. HX, DH, DL, GW, YJ, and LY performed the experiments. HX and DL wrote the first draft of the manuscript. SL, GW, YJ, XW, ZW, LB, RZ, HZ, LY, and ZG conducted data collection, statistical analysis, and wrote sections of the manuscript. DH, DL, RZ, HZ, LB, and JG contributed to manuscript revision. All authors read and approved the submitted version. All authors contributed to the article and approved the submitted version.

## Conflict of Interest

The authors declare that the research was conducted in the absence of any commercial or financial relationships that could be construed as a potential conflict of interest.
