# Supplementary material for: Bacteriophage Protects Against Aerococcus viridans Infection in a Murine Mastitis Model
Source: Front Vet Sci. 2020 Aug 28;7:588. doi: 10.3389/fvets.2020.00588 (PMC7485434; doi:10.3389/fvets.2020.00588)
Supplement: Supplementary file 1 [file Data_Sheet_1.docx]

**Supplementary files:**





**Figure S1**. The results of PCR (16S rRNA) with *Aerococcus viridans*–specific primers. lane M_1_ and M_2_ contain 2000-bp DNA marker; Lane 1-6 contain PCR fragments of M13-1, N14, N15, M6-1, M19-1 and P_1-1_F_2_, respectively.

**
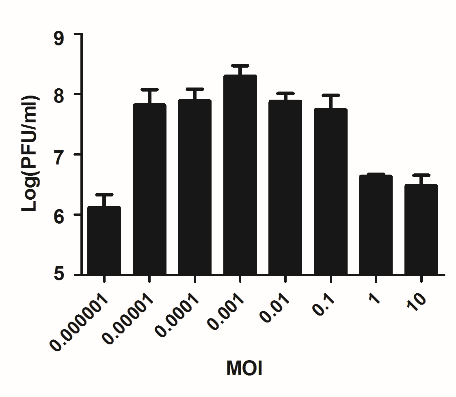
**

**Figure S2**. Titres of AVP under different multiplicities of infection (MOI). At MOI of 0.001, the AVP titres reached >10^8^ PFU/mL.

Table S1 Lytic activity of phage AVP against *Aerococci*

| Strain No.^a^ | Bacterial strain | Spot of AVP |
| --- | --- | --- |
| 1 | AV-X1 | + |
| 2 | M13-1 | + |
| 3 | N14 | + |
| 4 | N15 | + |
| 5 | Aer-2 | + |
| 6 | Aer-4 | + |
| 7 | Aer-5 | + |
| 8 | M6-1 | + |
| 9 | ATCC 51268 | - |
| 10 | CZ4b-3 | - |
| 11 | Aer-1 | - |
| 12 | Aer-3 | - |
| 13 | Aer-6 | - |
| 14 | P_1-1_F_2_ | - |
| 15 | M19-1 | - |

+: lysis -: no lysis
